# Frequent Recurrences of Genital Herpes Are Associated with Enhanced Systemic HSV-Specific T Cell Response

**DOI:** 10.1155/2020/5640960

**Published:** 2020-01-23

**Authors:** Michal Holub, Alžběta Stráníková, Pavel Chalupa, Simona Arientová, Kateřina Roubalová, Ondřej Beran

**Affiliations:** ^1^Department of Infectious Diseases, First Faculty of Medicine, Charles University and Military University Hospital Prague, U Vojenské nemocnice 1200, 169 02 Prague 6, Czech Republic; ^2^Department of Infectious and Tropical Diseases, First Faculty of Medicine, Charles University and Na Bulovce Hospital, Budínova 2, 180 81 Prague 8, Czech Republic; ^3^Institute of Haematology and Blood Transfusion, Department of Immunology, U Nemocnice 2094/1, 128 20 Prague, Czech Republic

## Abstract

**Objectives:**

Genital herpes simplex virus (HSV) infection is controlled by HSV-specific T cells in the genital tract, and the role of systemic T cell responses is not fully understood. Thus, we analysed T cell responses in patients with recurrent genital herpes (GH).

**Methods:**

T cell responses to HSV-1 and HSV-2 native antigens and the expression of HLA-DR and CD38 molecules on circulating CD8+ T cells were analysed in adults with high frequency of GH recurrences (19 patients) and low frequency of GH recurrences (7 patients) and 12 HSV-2 seronegative healthy controls. The study utilized the interferon-*γ* Elispot assay for measurement of spot-forming cells (SFC) after ex vivo stimulation with HSV antigens and flow cytometry for analysis of the expression of activation markers in unstimulated T cells.

**Results:**

The patients with high frequency of GH recurrences (mean number of recurrences of 13.3 per year) had significantly enhanced HSV-specific T cell responses than the HSV-2 seronegative healthy controls. Moreover, a trend of higher numbers of SFC was observed in these patients when compared with those with low frequency of GH recurrences (mean number of recurrences of 3.3 per year). Additionally, no differences in CD38 and HLA-DR expression on circulating CD8+ T cells were found among the study groups.

**Conclusions:**

Frequency of GH recurrences positively correlates with high numbers of systemic HSV-specific T cells.

## 1. Introduction

Genital herpes (GH) represents an important health problem. The disease is caused by herpes simplex virus (HSV) type 1 or type 2 entering the body through the genital tract of a nonimmune person. After the primary HSV infection, the virus stays dormant in the dorsal nerve root ganglia. In some individuals with latent infection, the virus reactivates several times yearly and the reactivation may lead to either recurrent GH or asymptomatic genital shedding of HSV-2 [1].

The latency of HSV in the dorsal nerve root ganglia is maintained by viral, cell, and immune mechanisms [2]. After the reactivation of virus, its replication is suppressed mostly by HSV-specific immunity. It has been postulated that HSV-specific CD8+ T cells are the most important immune cell types in providing immune surveillance in the tissues because in situ hybridization studies revealed their localization in latently infected ganglia and in the proximity of sensory nerve endings in the skin of genitalia of patients with recurrent GH [3,4]. Some studies also suggest a role of HSV-specific CD4+ T cells because genital ulcerations caused by HSV-2 are heavily infiltrated by this cell type [5].

Only scarce data are available on systemic HSV-specific T cell-mediated responses in the blood of patients with recurrent GH. A study of CD4+ T cell-mediated responses after ex vivo stimulation with HSV antigens did not demonstrate significant differences among patients with recurrent GH- and HSV-seropositive healthy controls [6]. In this study, the only significant difference in HSV-specific peripheral CD4+ T cell response was found between recurrent GH and HSV meningitis. Similarly, a study of the activation of circulating CD8+ T cells in HSV-2-infected individuals did not reveal significant changes in patients with recurrent GH [7]. On the other hand, a study of T-cell-mediated responses after primary HSV-2 infection reported persistent HSV-2-specific immune responses in a cohort with nonprimary genital HSV infection [8].

Therefore, we aimed to compare T cell-mediated HSV-specific responses after stimulation with either HSV-1 or HSV-2 native antigens in groups of patients with low and high frequency of GH recurrences and HSV-2 seronegative healthy controls. In addition, we compared the expression of activation markers on circulating CD8+ T cells among the study groups.

## 2. Patients and Methods

### 2.1. Patients

Twenty-six adults with HSV-2 infection were included in the study. All patients were enrolled at an outpatient clinic for chronic HSV infection, Department of Infectious and Tropical Diseases in the Na Bulovce Hospital, Prague, during the years 2011-2012. They were divided into two groups according to the frequency of GH recurrences: group 1 with a low frequency of GH recurrences (<10 recurrences yearly, mean of 3.3 recurrences per year) and group 2 with high frequency of GH recurrences (>10 recurrences yearly, mean of 13.3 recurrences a year). The etiology of GH was confirmed by the detection of HSV-2 DNA in genital ulcerations or by characteristic clinical findings of recurrent genital lesions responding to acyclovir therapy in a patient with positive anti-HSV-2 serology. The control group consisted of 12 HSV-2 seronegative healthy controls without a history of GH. The demographic, clinical, and laboratory data of the enrolled subjects are presented in Table 1. This prospective study was conducted in accordance with the Declaration of Helsinki after obtaining approval from the local ethics committee. A written informed consent was required from the study participants prior their enrolment.

### 2.2. Methods

#### 2.2.1. Serology

Specific antibodies were detected with chemiluminescence immunoassay tests for HSV-1/2 IgM, HSV-1/2 IgG, HSV-1 IgG, and HSV-2 IgG (DiaSorin, Saluggia, Italy) using a Liaison analyser (DiaSorin).

#### 2.2.2. Polymerase Chain Reaction

The specimens were obtained from the genital ulceration using Dacron swab FLOQswabs™ (Copan, Brescia, Italy) and processed on a Chemagic Prepito™ automatic analyser (PerkinElmer) using nucleic acid isolation with magnetic beads. The Chemagen Prepito Viral DNA/RNA200 kit was used for nucleic acid isolation. HSV DNA was detected by polymerase chain reaction (PCR) using the EliGene HSV-1 RT and EliGene HSV-2 RT tests (Elisabeth Pharmacon, Brno, Czech Republic) and an EliGene analyser ABI 7300™ (Applied Biosystems Inc, Foster City, CA, USA).

#### 2.2.3. Herpes Simplex Virus Native Antigens

HSV-1-CI native and HSV-2-CI native (Vidia, Vestec, Czech Republic) antigens were prepared as lysates from VERO cells infected with HSV-1 or HSV-2 (strain Praha and strain 910, respectively). When approximately 90% of the infected cells displayed a cytopathic effect, the cultures were harvested, washed with phosphate-buffered saline (PBS), and extracted with extraction buffer (0.01 M glycine, 0.1 M NaCl, pH 7.2, supplemented with the Protease Inhibitor Cocktail, Roche) for 2 hours at 37°C. Subsequently, the cell debris was diminished by centrifugation for 30 min at 1900 rcf, and the supernatant was lyophilized.

#### 2.2.4. Flow Cytometry

CD8+ T cell expression of CD38 and HLA-DR was measured in peripheral blood mononuclear cells (PMBC) using four-color flow cytometry and monoclonal antibodies against CD3, CD8, CD38, and HLA-DR molecules (BD Biosciences, San Jose, CA, USA). The data were analysed with BD FACSCanto II™ and BD FACSDiva™ software (BD Biosciences).

#### 2.2.5. Elispot Assays

The interferon (IFN)-*γ* Elispot assay was performed from cryopreserved PBMC, as previously described [9] with modifications. Wells were precoated using Capture Antibody Elispot Development Module Human IFN-*γ* (R&D Systems, Minneapolis, MN, USA). The cells were plated at 1 × 10^5^ PBMC per well in duplicate. HSV-1 and/or HSV-2 native antigens were added to the wells at a final concentration of 5 *μ*g/mL. Wells containing phorbol myristate acetate (PMA, 100 ng/mL) + ionomycin (1 *μ*g/mL) or CEF peptide mix of 9 mer–11 mer peptides from cytomegalovirus, Epstein–Barr virus, and influenza proteins (1.5 *μ*g/mL) served as positive controls. Wells containing RPMI served as negative controls. CD4 and CD8 T cell antigen-specific responses were expressed as spot-forming cells (SFC) per 10^6^ PBMC after subtraction of the mean patient background.

#### 2.2.6. Ethics Approval

The study was approved by the Ethics Committee of Na Bulovce Hospital (reference number 14.2.2011/5467/EK-Z).

#### 2.2.7. Statistical Analysis

Statistical differences among group 1, group 2, and the healthy controls were tested with the Kruskal–Wallis test (a nonparametric version of classical one-way ANOVA) using SigmaStat® software version 3 (Jandell Scientific, San Rafael, CA, USA).

## 3. Results

**Figure 1:**
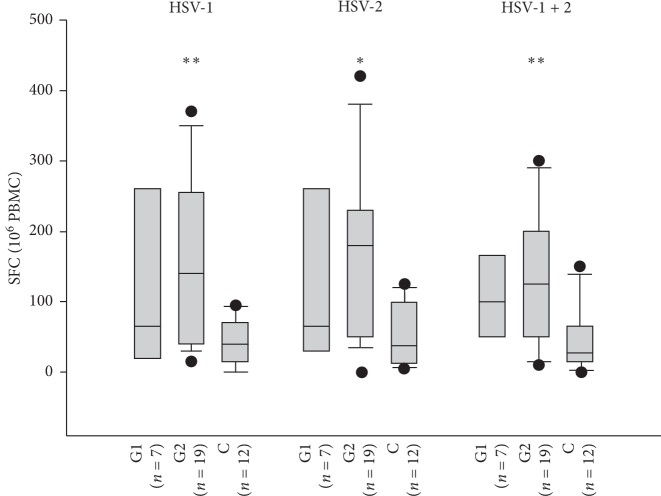
The comparison of HSV-1 and HSV-2-specific interferon-*γ* production measured by Elispot in patients with genital herpes and HSV-2 seronegative healthy controls (C). Patient groups: group 1 (G1), patients with <10 genital herpes recurrences per a year; group 2 (G2), patients with >10 genital herpes recurrences per a year. Interferon-*γ* production is reported as spot-forming cells (SFC)/10^6^ peripheral blood mononuclear cells (PBMC). The 25th and 75th percentiles define boxes, and the median values are indicated. The Kruskal–Wallis test was used to detect significant differences among the groups indicated above the boxes versus controls; ^*∗*^*p* < 0.05, ^*∗∗*^*p* < 0.01.

The results of laboratory analyses and the comparison among the groups are shown in Figure 1 and Table 2. HSV-specific T cell responses were significantly higher in patients with high frequency of GH recurrences (140 and 180 SFC for HSV-1 and HSV-2 stimulation, respectively; Table 2) than in the control group (40 and 37 SFC for HSV-1 and HSV-2 stimulation; *p* < 0.01 and *p* < 0.05, respectively). On the contrary, HSV-specific T cell responses in patients with low frequency of GH recurrences (65 and 65 SFC for HSV-1 and HSV-2 stimulation, respectively) did not significantly differ from that of the control group. The graphical comparison of SFC values in Figure 1 documents the differences among the patient groups and the control group. Moreover, the figure displays a trend in pronounced HSV-specific T cell responses in patients with high frequency of GH recurrence compared with patients with a low frequency of GH recurrences.

In contrast, surface expression of activation markers on circulating CD8+ T cells did not differ among the study groups. As shown in Table 2, the lack of difference was observed for the percentages of CD38+CD8+ T cells in patients with high frequency of GH recurrences, low frequency of GH recurrences, and the control group (8.0%, 9.3% and 9.1%, respectively). Similarly, the percentages of HLA-DR+CD8+ T cells did not differ among patients with high frequency of GH recurrences, low frequency of GH recurrences, and the control group (6.3%, 2.6% and 7.0%, respectively). This suggests that the expression of markers of chronic activation on circulating immune cells is not associated with GH reactivations.

## 4. Discussion

In this study, we evaluated HSV-specific T cell responses and the expression of immune activation markers on peripheral CD8+ T cells in blood from patients with recurrent GH. The IFN-*γ* Elispot assay was used because it has high sensitivity for the detection of specific T cells and is simple and reproducible [10]. We observed a positive correlation of HSV-specific T cells with frequency of GH recurrences; however, the recurrences were not associated with changes in expression of activation markers on peripheral CD8+ T cells.

It is well known that virus-specific systemic immune responses are not always effective in the suppression of viral infection. Moreover, the development of chronic infection is frequently accompanied by specific and nonspecific immune activation. A good example is human immunodeficiency virus (HIV) infection, which is associated with strong systemic T cell responses despite the disease progression [11,12]. Similar to HIV, we observed increased levels of HSV-specific T cells in the blood from patients with high frequency of GH recurrences. However, in contrast to HIV infection, the signs of systemic immune activation (i.e., elevated expression of CD38 and HLA-DR molecules on CD8+ T cells) were not present in patients with recurrent GH. This discrepancy may be explained by different characters of HIV and HSV infections: HIV infection has a generalized course and is accompanied by peripheral T cell activation, not only due to massive immune stimulation by high levels of the virus antigens in peripheral blood but also because of their immunostimulatory properties [13]. On the other hand, in GH, HSV infection is mostly localized in the immuno-privileged genital tract and elicits local immune response with minimal impact on the activation of immune cells in the periphery [14]. A suppressive effect of HSV-2 on systemic immune activation was observed in patients with HSV-2 and HIV co-infection [7]. High levels of HSV-specific T cells in the peripheral blood of the patients with high frequency of GH recurrences in our study may be associated with repeated exposure to viral antigens. Similar results were presented by Franzen-Röhl et al. in a prospective study of HSV-2 recurrence after primary infection [8]. Additionally, in the extensive prospective study conducted by Moss et al. [15], the authors did not find a correlation between the frequency of viral shedding or frequency of GH recurrences and the magnitude of HSV-specific CD4+ T cell response. In our assay, we measured total T cell response to HSV antigens, including not only CD4+ but also CD8+ T cells, that were previously identified as a major component of cellular immune response to herpes virus infection [16]. Nevertheless, our data may support the view that the rate of HSV-specific peripheral T cell response reflects repeated antigenic stimulation rather than the level of immunosurveillance of the virus latency. The latter seems to be provided mainly by HSV-specific CD8+ T cells localized in latently infected ganglia [4,17].

We observed that the specific T cell responses induced by HSV-1 and HSV-2 native antigens were similar even though the vast majority of patients had the recurrent GH caused by HSV-2. Moreover, the T cell responses after stimulation with HSV-1 native antigens in seven patients with GH with anti-HSV-1 negative serology did not differ from the HSV-1 seropositive study subjects (data not shown). These findings can be explained by the cross-reactivity of HSV-1 and HSV-2 viral antigens containing both the type-specific and type-common antigenic determinants. Alternatively, some HSV-infected individuals may mount a specific cellular immune response in the absence of detectable HSV-specific antibodies [18]. On the other hand, neither the magnitude of HSV-2-specific cellular immune responses nor the titres of specific antibody were associated with the acquisition of HSV-2 infection by vaccinated individuals [19]. Taken together, these findings support the notion that, at present, there are no available correlates of protective immunity against either primary or recurrent HSV-2 infection [20].

Our study has several limitations. First, the stimulation of T cells with HSV-1 or HSV-2 native antigens is not an optimal approach because both viruses share common antigenic determinants [21]. Moreover, it was shown that native viral antigens ex vivo stimulate mostly CD4+ T cells, while CD8+ T cell stimulation is not very effective [22]. On the other hand, the use of HSV native antigens has some advantages over the use of specific viral peptides because it eliminates differences in immunodominance among different peptides, does not depend on HLA specificity, and contains many adjuvant Toll-like receptor ligands that increase the effectiveness of antigen presentation [23]. Second, the diagnosis of HSV-2 infection was confirmed by the detection of HSV DNA in genital ulceration only in 18 of 26 enrolled subjects. The remaining eight patients had a diagnosis of recurrent GH of HSV-2 etiology established based on characteristic clinical findings of recurrent genital ulcerations responding to oral acyclovir therapy and positive HSV-2 serology. Thus, we cannot exclude the possibility that some cases of recurrent GH were caused by HSV-1. However, the number of GH recurrences in these patients was relatively high, which supports HSV-2 as the cause of recurrent GH because HSV-1 genital infection is rarely associated with the recurrent disease [24,25].

## 5. Conclusion

These findings indicate that high numbers of systemic HSV-specific T cells are not a marker of immune control of latent genital HSV infection. However, the immune responses in the genital compartment can be totally different. Therefore, studies aimed at HSV-2-specific immunity in the genital tract are warranted, because better understanding of immune mechanisms leading to suppression GH recurrences is necessary for successful development of efficient therapeutic vaccines.

## Figures and Tables

**Table 1:** Demographic, clinical, and laboratory data of patients with genital herpes and HSV-2 seronegative healthy controls.

Parameter	Group 1 (*n* = 7)	Group 2 (*n* = 19)	Controls (*n* = 12)
Sex (male/female)	0/7	7/12	4/8
Age (mean, range)	48.7, 21–73	39.2, 27–60	35.8, 26–61
Recurrences (mean, range)	3.3, 2–6	13.3, 10–24	—
Detection of HSV DNA in genital swab (+/0)	4/3	14/5	—
Presence of anti-HSV-2 IgG (+/−)	7/0	17/2	0/12
Presence of anti-HSV-1 IgG (+/−)	5/2	12/7	8/4
Suppressive therapy (valaciclovir)	1	6	—
Episodicall treatment (valaciclovir or acyclovir)	4	13	—
Without treatment	2	—	12

Group 1, patients with low frequency of GH recurrences (<10 recurrences/year); Group 2, patients high frequency of GH recurrences (>10 recurrences/year); +, positive; −, negative.

**Table 2:** Comparison of immunological parameters in patients with genital herpes and HSV-2 seronegative healthy controls.

Parameter	Group 1 (*n* = 7)	Group 2 (*n* = 19)	Controls (*n* = 12)	*p* value
CD8+CD38+T cells (%; range)	9.3; 8.7–9.9	8.0; 6.9–9.9	9.1; 6.4–1.1	NS
CD8+HLA-DR+T cells (%; range)	2.6; 2.3–1.2	6.3; 5.0–8.1	7.0; 4.0–1.0	NS
CD8+CD38+HLA-DR+T cells (%; range)	1.4; 1.2–5.0	2.3; 1.3–2.7	1.9; 1.1–4.3	NS
IFN-*γ*+T cells (SFC, HSV-1 stimulation)	65; 29–224	140; 48–249^*∗*^	40; 15–70	<0.01
IFN-*γ*+T cells (SFC, HSV-2 stimulation)	65; 34–243	180; 56–228^*∗*^	37; 15–98	<0.05
IFN-*γ*+T cells (SFC, HSV-1 + 2 stimulation)	100; 54–150	125; 54–199^*∗*^	27; 15–65	<0.01

Group 1, patients with low frequency of GH recurrences (<10 recurrences/year); Group 2, patients with high frequency of GH recurrences (>1024 recurrences/year); SFC, spot-forming cells/10^6^ peripheral blood mononuclear cells (mean; range); ^*∗*^significant difference from controls; NS, nonsignificant.

## Data Availability

Requests for data, 12 months after initial publication, will be considered by the corresponding author.

## Additional Points

*Key Messages*. (i) The high frequency of genital herpes recurrences positively correlates with the enhanced ex vivo T cell responses to HSV-1 and HSV-2 native antigens. (ii) T cells from patients with recurrent HSV-2 genital herpes react to HSV-1 and HSV-2 native antigens with similar frequencies. (iii) Recurrent genital herpes is not associated with increased activation of peripheral blood CD8+ T cells.
